# Speed Up of Volumetric Non-Local Transform-Domain Filter Utilising HPC Architecture

**DOI:** 10.3390/jimaging9110254

**Published:** 2023-11-20

**Authors:** Petr Strakos, Milan Jaros, Lubomir Riha, Tomas Kozubek

**Affiliations:** IT4Innovations, VSB—Technical University of Ostrava, 17. Listopadu 2172/15, 708 00 Ostrava-Poruba, Czech Republic

**Keywords:** volumetric data, image denoising, parallel implementation, medical imaging, high-performance computing

## Abstract

This paper presents a parallel implementation of a non-local transform-domain filter (BM4D). The effectiveness of the parallel implementation is demonstrated by denoising image series from computed tomography (CT) and magnetic resonance imaging (MRI). The basic idea of the filter is based on grouping and filtering similar data within the image. Due to the high level of similarity and data redundancy, the filter can provide even better denoising quality than current extensively used approaches based on deep learning (DL). In BM4D, cubes of voxels named patches are the essential image elements for filtering. Using voxels instead of pixels means that the area for searching similar patches is large. Because of this and the application of multi-dimensional transformations, the computation time of the filter is exceptionally long. The original implementation of BM4D is only single-threaded. We provide a parallel version of the filter that supports multi-core and many-core processors and scales on such versatile hardware resources, typical for high-performance computing clusters, even if they are concurrently used for the task. Our algorithm uses hybrid parallelisation that combines open multi-processing (OpenMP) and message passing interface (MPI) technologies and provides up to 283× speedup, which is a 99.65% reduction in processing time compared to the sequential version of the algorithm. In denoising quality, the method performs considerably better than recent DL methods on the data type that these methods have yet to be trained on.

## 1. Introduction

Medical imaging is a technical area where image processing plays a crucial role. It can considerably influence the quality of resulting visualisations. A significant level of noise typically corrupts the image data. Thus, image filtering is important here. The noise source is connected with the physical principles of the imaging methods. The commonly used medical imaging methods are computed tomography (CT), magnetic resonance imaging (MRI) and ultrasound. Powerful denoising techniques have to be used to reduce the noise level. There is a large field of techniques that focus on image denoising. The traditional ones represented by Gaussian filters [1], wavelet filters [2] and Wiener filters [3] are accompanied by methods using non-local means [4,5] that use non-local data to perform the filtering at specific locations within the image. Originally, the traditional techniques were designed to handle 2D images. However, this is less effective since most medical image data, i.e., CT and MRI, are 3D. For this purpose, the filtering techniques have been redesigned to fully utilise the volumetric nature of the data. Three-dimensional modifications of the non-local means filter can be found in [6,7,8,9]. A more powerful technique designed for filtering volumetric data that brings the state-of-the-art in the classical filtering methods is called BM4D. A detailed description of the method is presented in [10].

There is also enormous attention given to deep learning (DL) methods, which have proved effective in tasks such as image denoising. For example, [11] uses residual learning and deep convolutional neural networks to achieve state-of-the-art performance in image denoising. This method is not dedicated to medical image data but is proposed as a general image denoiser. On the other hand, the technique in [12] is used to reduce noise, specifically in low-dose CT images, and reaches the state-of-the-art in both simulated and clinical cases. It uses a residual encoder–decoder convolutional neural network to create the denoising algorithm. There are also quite popular DL-based techniques for image denoising that incorporate the image rendering process [13,14]. They are trained on large amounts of synthetic data and reduce the noise produced by Monte Carlo rendering methods like path tracing. However, based on [15], where authors evaluate the DL-based approach to minimise the noise in CT images with variable X-ray dose levels, it is observed that DL methods cannot always improve the image quality compared to traditional methods, and it is dependent on the type of data that the method is trained on and on specific noise levels that it is supposed to reduce. For example, the DL method trained only on general images performed better at denoising CT images corrupted by small noise levels than the same method fine-tuned on CT images with various noise levels. Opposed to that, the generally trained DL method performed poorly at denoising high noise levels. In contrast, the fine-tuned method could provide good results, but only at specific high noise levels, even though it was fine-tuned on CT data with all levels of corrupting noise. Although DL methods are frequently used, traditional image-denoising methods still have their place, and they can compete with DL-based methods, especially if there are not enough data to train the DL methods on or there are no data at all, which is the case of medical imaging. No ideal noise-less CT or MRI reference could be acquired from the measurement. Those could be only simulated.

**Figure 1:**
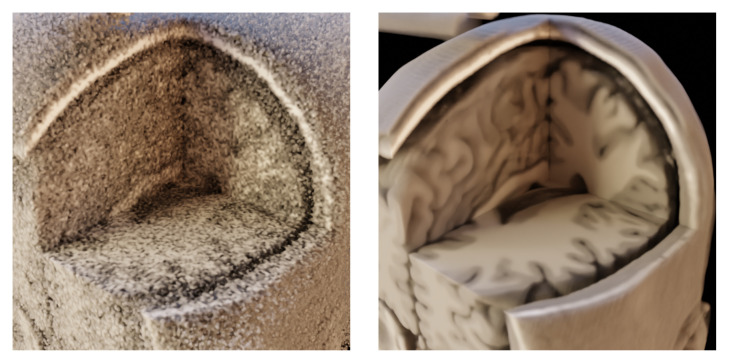
Volume rendering of MRI dataset from BrainWeb; (**left**)—the dataset corrupted by 25% Gaussian noise; (**right**)—the dataset without the noise; identical setup of a transfer function in shader properties for both data.

Our motivation is to provide a medical image denoising method that does not require training or fine-tuning on specific data due to the potential lack of data or their complete unavailability. On the other hand, such a method should still provide state-of-the-art denoising performance and fast computation times. This method would then pre-process the medical image data before volume rendering. In Figure 1, we show volume renders of the MRI dataset obtained from BrainWeb for noisy and noise-less data. The idea behind using a suitable denoising method is to provide similar visual quality as the volume rendering of reference noise-less data if we use the noisy input and image denoising before it is rendered.

We focus on the BM4D method, which is an extension of the BM3D filter described in [5], but instead of a group of pixels as used in 2D images, it works with cubes of 3D voxels. By working with voxels, the computational complexity rises. This is natural since more data are being processed. Unfortunately, the computational load can be so high that it makes using the filter very impractical. As documented in [10], to filter the image of size 181 × 217 × 181 voxels while using the proposed modified parameters of the filter, the total filtering time takes more than 11 min (676.7 s) on a machine with a 2.66 GHz processor and 8 GB of RAM. This is too much to make the filter practically available. The proposed filter implementation, as provided in [16], does not leverage parallel processing to reduce the computation time. The only parallel implementation of the BM4D filter we know of is in [17] and provides tests on multi-core and multi-node CPU resources only.

This paper presents an extension of work in [17] by describing and evaluating the parallel multi-core and new many-core implementation. Special attention is drawn to utilising Intel’s MIC (Intel Corporation, Santa Clara, CA, USA) (Many Integrated Core) architectures, either solely or in combination with Intel’s CPUs. We test our implementation on three clusters built on different HW, proving our solution’s universality. Our implementation is provided as a C++ code, using hybrid parallelisation via MPI and OpenMP frameworks. We evaluate the filter on three different datasets with two medical modalities, the MRI and the CT. The MRI data are from the BrainWeb database [18], which offers simulated brain data. Since the reference image is available in this dataset, we also compare our implementation with selected DL methods in denoising quality here. The other two CT modalities represent the data of real patients. The CT data are in a much larger image series than the one from BrainWeb. Selected datasets also evaluate the filter implementation in terms of different workloads. The contributions of the paper are as follows,

- Application of parallel version of BM4D algorithm to many-core architectures and combination of multi and many-core HW and proving the scalability tests.
- Comparison of the algorithm with DL-based approaches.
- Testing the algorithm as a pre-processing stage before volume rendering.

The paper is organised as follows. In Section 2.1, we briefly explain the basic idea of the BM4D algorithm. Section 2.2 describes the parallelisation concept that allows for running the algorithm in parallel instead of sequentially. Section 2.3 describes the programming techniques and tools used to make the algorithm run in parallel. Section 2.4 brings the information about the type of hardware that the algorithm has been tested on and the data used to test it. All the tests and respective results are provided in Section 3. Our conclusions and future work are presented in Section 4.

## 2. Materials and Methods

### 2.1. Explanation of BM4D Algorithm

A detailed explanation of the BM4D algorithm can be found in [10]. Here, we restrict ourselves to the basic idea of the algorithm and its workflow. It is later used to explain our way of parallelisation.

**Figure 2:**
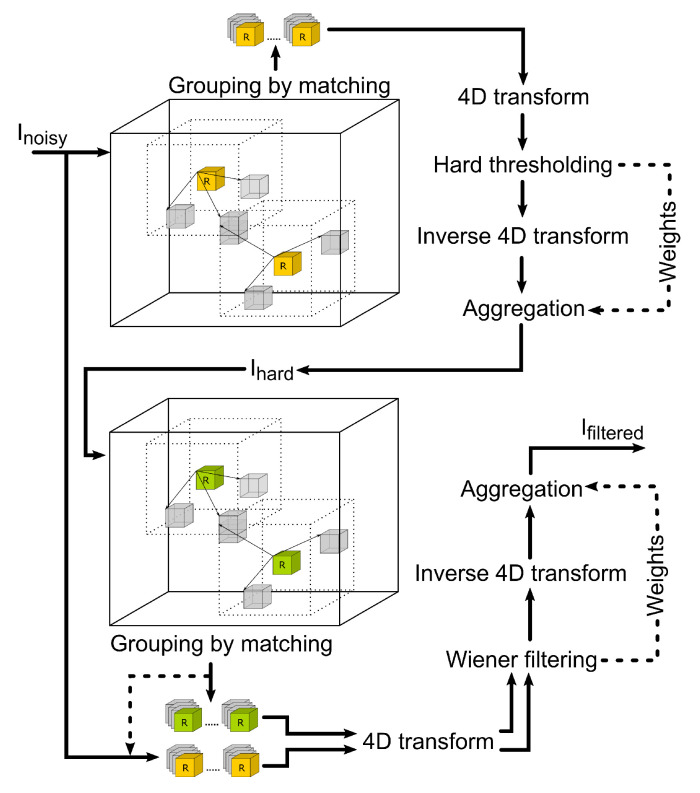
Workflow of the collaborative filtering method graphically describing the two subsequent steps—hard thresholding and Wiener filtering.

In Figure 2, the concept of the BM4D filter is shown. The whole procedure can be divided into two subsequent steps: hard thresholding of the image and Wiener filtering. In the first step, the noisy image in a 3D matrix is marched through, and reference cubes of voxels are selected. Around each reference cube, an area of specific size and cubical shape is set. Within this area, mutually similar cubes to the reference are searched. The most similar ones are stacked in a group. This procedure is called grouping by matching. Groups of similar cubes are then transformed into sparse domains. To transform the data, a 4D transformation is applied (it accounts for three spatial dimensions: x, y, z and one dimension in the order of the stacked cubes in the group). The resulting 4D transform is achieved by 4× 1D transform in respective order. This is also the approach that we use in our implementation; see the Algorithm 1, which, in detail, shows how the 4D transformation is achieved. After transforming into the sparse domain, the separation of noise and a valid signal is carried out effectively by hard thresholding. The filtered spatial data are obtained using the inverse 4D transform of the thresholded groups. Those are then aggregated in their original locations within the image. This provides the filtering in the first step of the algorithm.
**Algorithm 1:** Four-dimensional transformation from spatial to sparse domain.
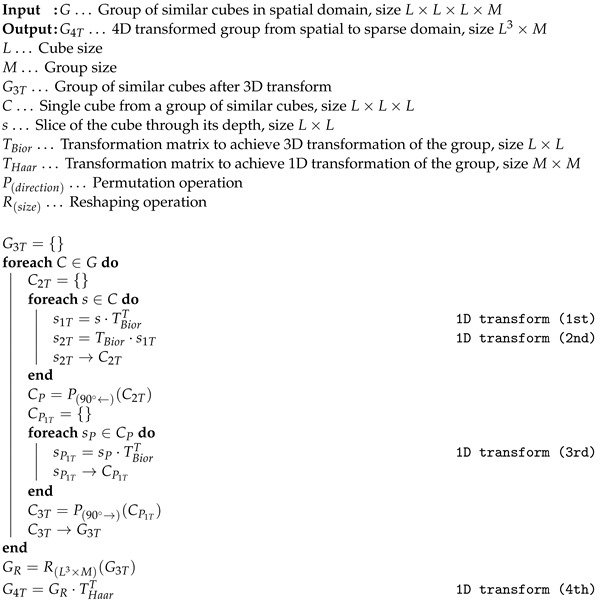


The second step uses the hard thresholded image and performs another grouping by matching. Because the noise in the image is already significantly reduced by previous operations, the grouping and matching can perform considerably better this time. Locations of the grouped cubes of voxels are used to collect similar groups within the original noisy image. Both groups are again transformed into the sparse domain, and the Wiener filtering is applied to the original noisy data. These data are then back-transformed and aggregated to form the final filtered image.

The BM4D algorithm works with several parameters that influence the speed and quality of the filtering. We have adopted the parameters recommended in [10] that are indicated as “modified profile“. This setting represents an assortment of optimised parameters that assure balanced performance in a wide range of noise variances. In [10], it is documented as having the best PSNR performance compared to the stated “normal profile”. Table 1 presents the modified parameters because we reference them further in the text.

### 2.2. Parallelisation Concept

**Figure 3:**
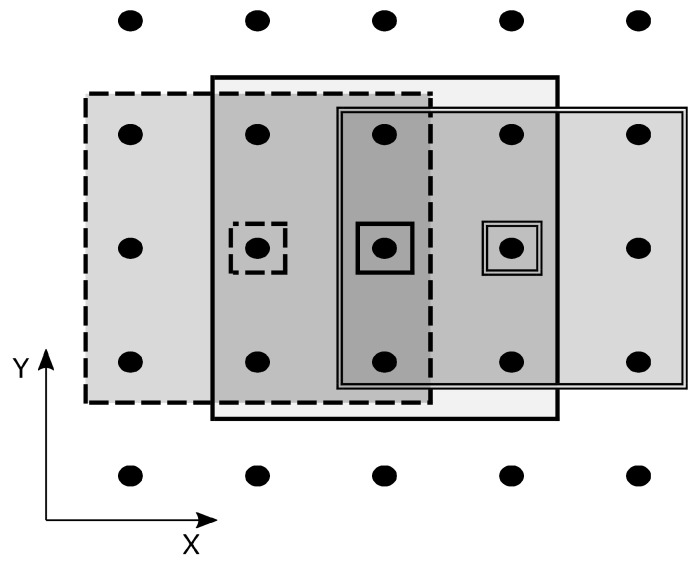
Sorting areas (large rectangles) around the reference patches (small rectangles) into groups with non-overlapping areas. One-dimensional example (X direction).

Parallelisation generally tries to leverage those parts of an algorithm that can run independently of the others. Independent parts of code can run in parallel on multiple threads and nodes and can therefore run much faster. In our approach, we work with the idea that the searched areas around each reference cube in the image can create completely separate tasks. This is possible if we can guarantee that the areas do not overlap. However, we had to resolve this issue because the BM4D filter uses overlapping areas to filter the whole image. The solution here is to sort the reference cubes with their respective areas into groups containing only areas that do not overlap. A one-dimensional example (X direction) of such a procedure is shown in Figure 3, where areas (outlined large rectangles) around each reference patch (small rectangle with black dot inside) are sorted into three different groups to preserve non-overlapping. A similar procedure is handled in all three directions (X, Y, Z). This can be applied inside each step, either hard thresholding or Wiener filtering. In this way, we can parallelise the most computationally extensive parts of the algorithm. Different groups have to be solved sequentially to preserve non-overlapping or total independence between concurrent threads. However, this is not a big issue since the bigger the image, the higher the amount of non-overlapping areas in a group that can be solved in parallel. Typically, the number of non-overlapping areas is much higher than the number of used parallel threads, so even within one group, the allocated resources are used more than once.

### 2.3. Practical Realisation of the Parallel Filter Implementation

We have used the Blender (Blender Foundation, Amsterdam, Netherlands, version 3.6) [19] environment to practically realise the filter implementation. We execute our BM4D filter in parallel using the server and client(s) approach. Blender is open-source software and serves as a visualisation environment where we can load datasets, process them and visualise them. The Blender environment is optional since the concept of server and client(s) is general and fits into the rules of MPI communication, which we use to utilise multi-node resources. Our concept uses hybrid parallelisation that exploits both MPI [20] and OpenMP [21] frameworks. In this way, we can effectively use the computational resources of a multi-core workstation or even a multi-node supercomputer. We also paid special attention to creating the filter implementation as general enough so it can utilise different types of architectures typical in supercomputers; see Section 2.4. Our concept can use cluster nodes fully, whether equipped with multi-core (CPU) or many-core (MIC) processors or combinations.

**Figure 4:**
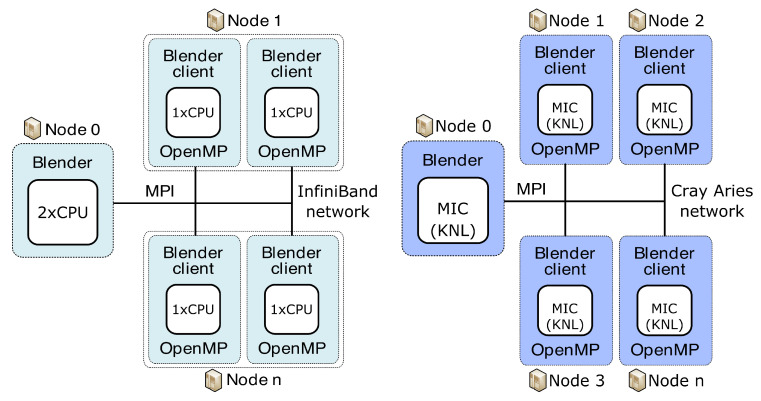
Concept of server and clients used for parallelisation of BM4D algorithm on multiple CPU nodes of Anselm (**left**) and multiple MIC nodes of HLRN’s test system (**right**).

**Figure 5:**
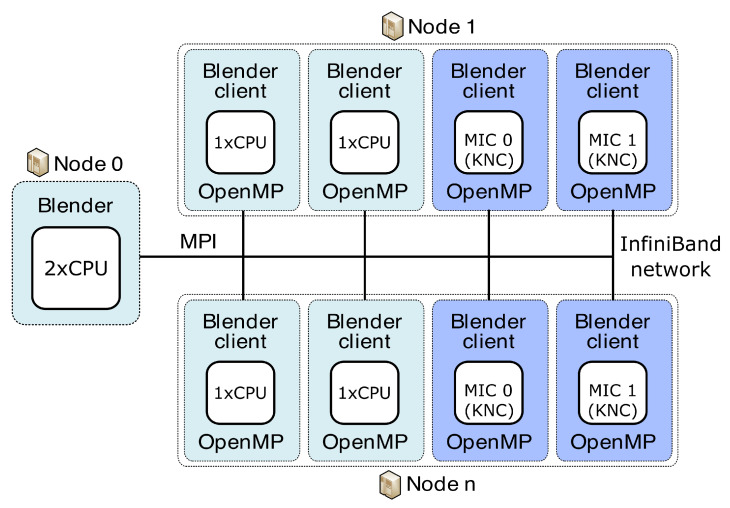
Concept of server and clients used for parallelisation of BM4D algorithm on multiple CPU/MIC nodes of Salomon.

Our concept of the server and clients used to run on different clusters is organised as shown in Figure 4 and Figure 5. Blender runs on the server and collects results from individual clients previously given a specific portion of the total workload. As a server, we can use either the classical CPU node or the second-generation MIC node (KNL, aka “Knights Landing”) (Intel Corporation, Santa Clara, CA, USA). Blender clients can run on classical CPUs or both generations of MIC, specifically the first-generation KNC (aka “Knights Corner”) or the second-generation KNL. Before sending specific workloads to clients, the pre-processing phase consists of the creation of transformation matrices and division into separable and balanced workloads. This is performed on the server side. All of the necessary data needed for the job on each client are then distributed from the server using collective MPI routine *MPI_Broadcast*. We broadcast the image data, the transformation matrices and the vector of groups of reference cube’s coordinates. Each group in the vector guarantees that all coordinates in one group point to the mutually non-overlapping areas in the image. In such a way, those image parts can run in parallel when computed on a specific client. Each client locally utilises the OpenMP’s *#pragma* directives for parallelisation. We use *#pragma omp parallel for schedule(guided)* to best tackle the possible load imbalance between iterations of the parallel loop. After each node finishes work, the server collects partial results over the image using another collective MPI routine, the *MPI_Reduce*. We use the *MPI_SUM* operation in *MPI_Reduce* to sum up the portions of the numerator and denominator that are part of the aggregation process in the BM4D algorithm. By using collective routines, we can reduce the communication overhead due to tree-based optimisation while communicating. We also use a strategy aware of the NUMA (non-uniform memory access) effect on CPUs. Within one node, we access only that part of CPU memory dedicated to a specific processor, meaning that the memory access is uniform. This feature can bring more than 20% speed up (when measured on 15 clients). Practically, it means that we attach one client to exactly one processor; see Figure 4 and Figure 5.

### 2.4. Utilised HPC Resources and Test Data

We have tested the parallel implementation of the BM4D filter on three different supercomputers equipped with different hardware. Clusters offer typical multi-core processor nodes (CPUs), purely many-core processor nodes (MICs), or their combination. The first one, Salomon [22], is a multi-node cluster where each regular compute node is an x86-64 computer equipped with twenty-four cores (two twelve-core Intel Haswell processors). There are also accelerated nodes that, besides the regular ones, have two many-core Intel Xeon Phi 7120P accelerators (KNCs) on each node. The second supercomputer, Anselm [23], is Salomon’s predecessor, and it is equipped with sixteen cores (two eight-core Intel Sandy Bridge processors) on each of its regular nodes. The third cluster is HLRN’s Cray test and development system [24] that is equipped with many-core processors, Intel Xeon Phi 7250 (KNL). One processor is dedicated to one node on this system.

**Figure 6:**
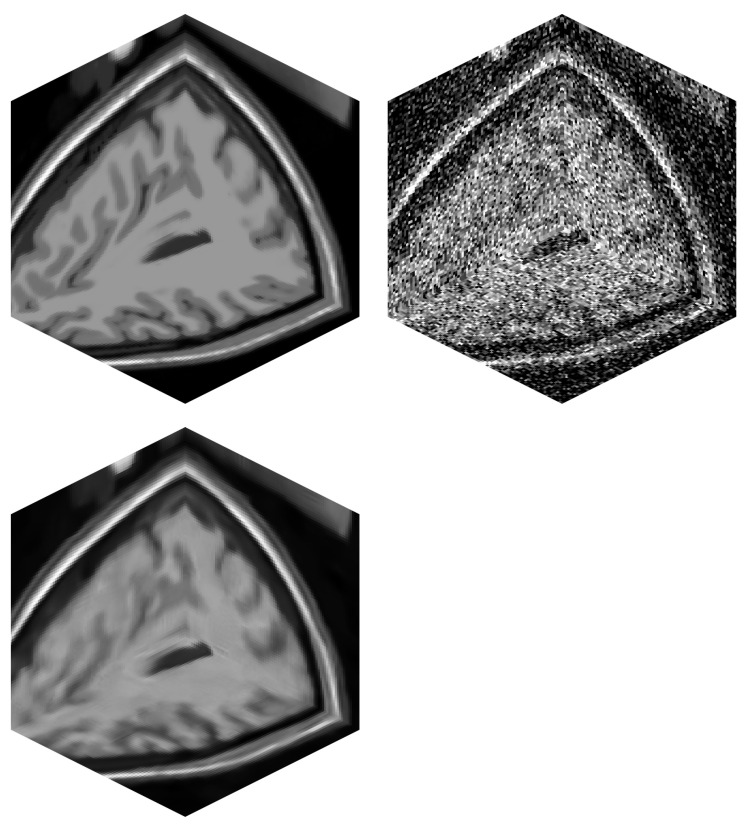
MRI volume of a brain (BrainWeb). (**top left**)—Original noise-less data. (**top right**)—Original data corrupted by 25% Gaussian noise. (**bottom left**)—Filtered image data by BM4D.

**Figure 7:**
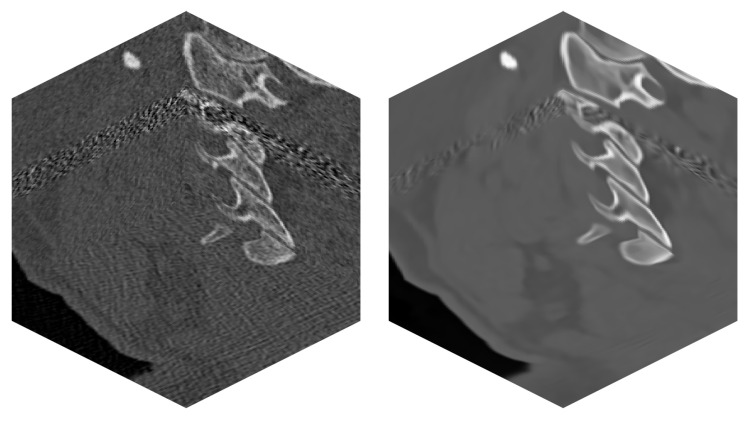
Real CT data of a patient—series of 170 images. (**left**)—Original noisy data. (**right**)—Filtered image data by BM4D.

**Figure 8:**
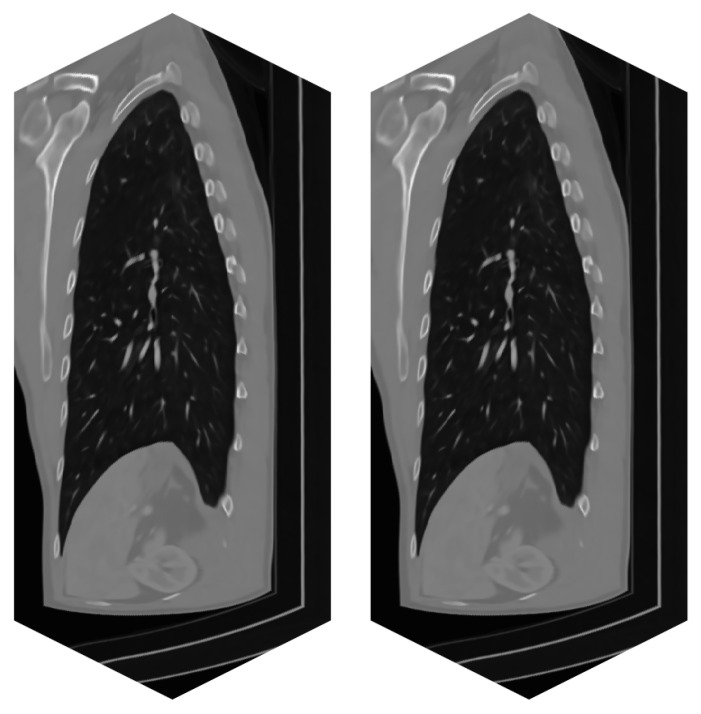
Real CT data of a patient—series of 779 images. (**left**)—Original noisy data. (**right**)—Filtered image data by BM4D.

The tests were run on three different datasets. They differ in size and modality. The smaller dataset of size 181 × 181 × 181 voxels represents simulated MRI brain data; see Figure 6. The larger dataset of size 512 × 512 × 170 voxels is the CT modality, and it covers the head–neck area of an actual patient; see Figure 7. The largest dataset has the size of 512 × 512 × 779 voxels, and it is again a CT scan of an actual patient. This dataset covers the patient’s thorax and abdomen; see Figure 8. Selected datasets vary also in terms of voxel dimensions and proportions. The 512 × 512 × 779 dataset has voxel size [0.71, 0.71, 0.6] mm, while voxels in the 512 × 512 × 170 dataset are size [0.38, 0.38, 0.75] mm. The 181 × 181 × 181 dataset uses voxel size [1, 1, 1] mm. These specific datasets were chosen to stress out the proposed algorithm in possible scenarios, such as processing isotropic and anisotropic voxels, computing on different data modalities (CT, MRI) and using small to large data sizes.

## 3. Results

**Figure 9:**
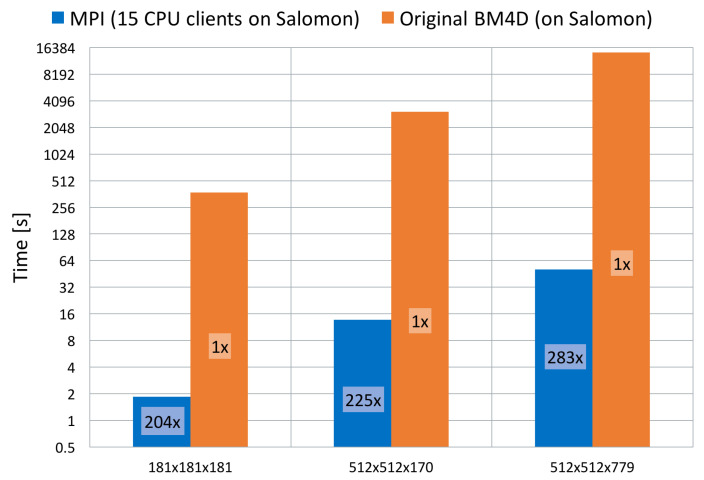
Comparison between original BM4D [16] and our parallel implementation in terms of total runtime and achieved speed-up. Results shown on all three data sets.

At first, we have compared results obtained by the original single-threaded algorithm implementation [16] as a non-parallelised version of the code with our parallelised version. This measurement has been performed on all three datasets to show and fully appreciate the effect of the parallelisation. Results can be seen in Figure 9.

For further tests of algorithm scalability, we have used allocation of up to 32 nodes depending on the cluster that we are testing on (32 nodes when testing on HLRN’s cluster and 17 nodes of Salomon or Anselm). It is a reasonable amount for measuring the filter implementation. Measurements have been conducted on the biggest dataset of 512 × 512 × 779 voxels.

**Figure 10:**
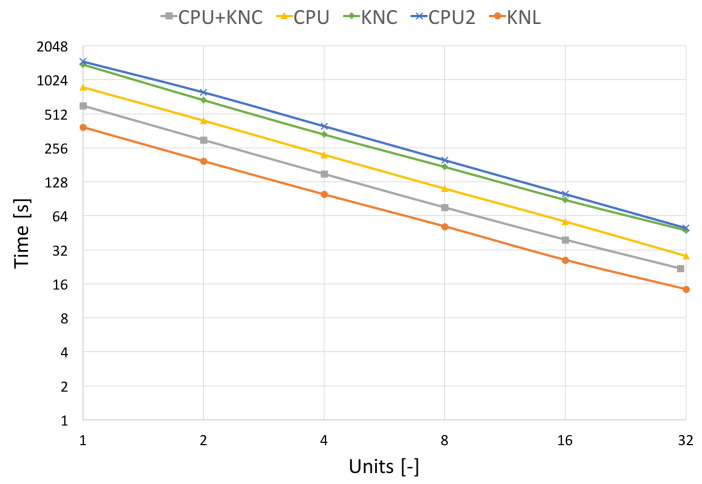
Strong scalability on different architectures **without** communication. Results on the real CT data of size 512 × 512 × 779 voxels.

**Figure 11:**
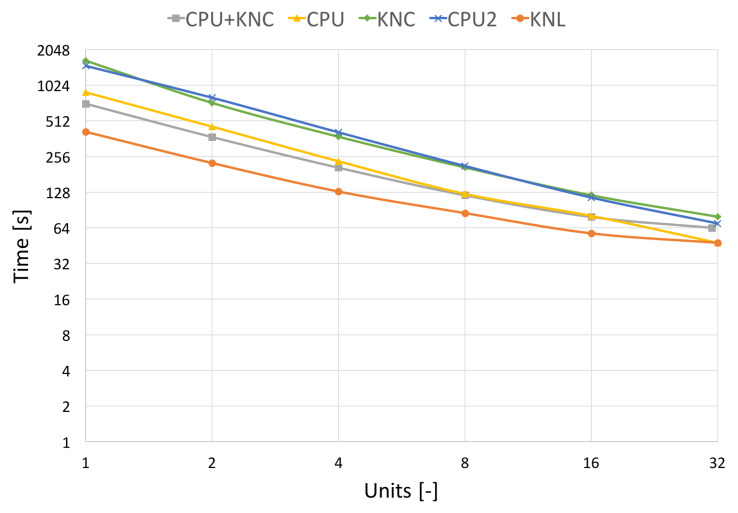
Strong scalability on different architectures **with** communication. Results on the real CT data of size 512 × 512 × 779 voxels.

In Figure 10 and Figure 11, which capture the scalability tests, we show two processing times, the pure filtering time and the total runtime. The total algorithm runtime includes the pre-processing time (assembly of transformation matrices, division to groups of non-overlapping areas, etc.), the communication time used by MPI and the time spent by filtering the image. The filtering time can be further divided into the time spent by hard-thresholding and the time spent by Wiener filtering.

A significant result that is coming out of the tests is a speed comparison of different generations of utilised HW. We have used two generations of Intel’s MIC (KNC and KNL) and two of Intel’s CPUs (Haswell and Sandy Bridge). Haswell can be compared with KNL (introduced in 2013) and Sandy Bridge with KNC (introduced in 2011). We have always attached one CPU to one MPI process (one Blender client). This means 12 CPU cores of Salomon as one process and 8 CPU cores of Anselm as one process. These can be compared to one KNL MIC or one KNC MIC, respectively.

To compare the different HWs, we have also selected the term “Units”, which stands for specific resource utilisation on different HWs. On HLRN’s cluster, one unit stands for utilising one KNL. A similar case holds for the Anselm cluster, where one unit is one CPU. Since the Salomon cluster is equipped with CPUs and KNCs within one node, we distinguish based on their combination used for testing. If CPU and KNC are utilised, then one unit stands for one CPU and one KNC. If only a CPU is used, then one unit is one CPU. A similar case holds if only KNC is used; then, one unit stands for one KNC.

Figure 10 shows the pure filtering time obtained on different HW. Here, the CPU+KNC stands for measurement on Salomon utilising all resources within the nodes, CPU stands for only CPU utilisation on Salomon, KNC stands for only KNC utilisation on Salomon, CPU2 uses the CPU nodes of Anselm and KNL utilises the KNL nodes of HLRN cluster. The results here show almost ideal scalability since they focus on algorithm speed up without the communication burden.

Figure 11 shows the total algorithm runtime, utilising the same resources as in the previous Figure 10. We can see a decline in scalability due to the high communication burden of MPI. This effect affects the MIC processors more in the case of pure KNL, KNC or a combination of CPU+KNC.

As for the comparison of different HW, it is shown that the Sandy Bridge architecture (1×CPU of Anselm) is comparable with MIC KNC architecture (1×KNC of Salomon), whilst Haswell architecture (1×CPU of Salomon) lags behind MIC KNL architecture (1×KNL of HLRN’s).

**Figure 12:**
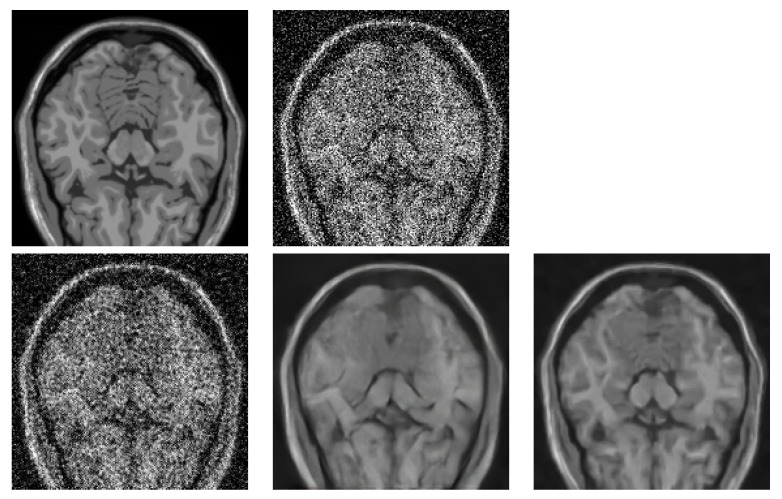
Visual comparison of different denoising methods applied on BrainWeb dataset (181 × 181 × 181 voxels). Image from axial slice 56 is used for comparison. (**top left**)—Original noise-less data. (**top right**)—Original data corrupted by 25% Gaussian noise. (**bottom left**)—RED-CNN. (**bottom middle**)—OIDN. (**bottom right**)— BM4D.

In the next tests, we compared the BM4D denoising quality with two DL-based methods, specifically with RED-CNN [12] and OIDN [13]. The former is a method specialising in noise reduction in low-dose CT images. The latter is a very popular method used for general image denoising, and it is typically used for noise reduction in ray-traced images. We have used this method specifically because we also perform volume rendering tests in the last step. None of the DL methods have been fine-tuned on the MRI data. We wanted to see their raw potential. We show the denoising quality comparison in Table 2. The three metrics, peak signal-to-noise ratio (PSNR), structural similarity index measure (SSIM) and root mean square error (RMSE), were chosen for the image quality assessment. A visual comparison of the respective method performance is provided in Figure 12.

**Figure 13:**
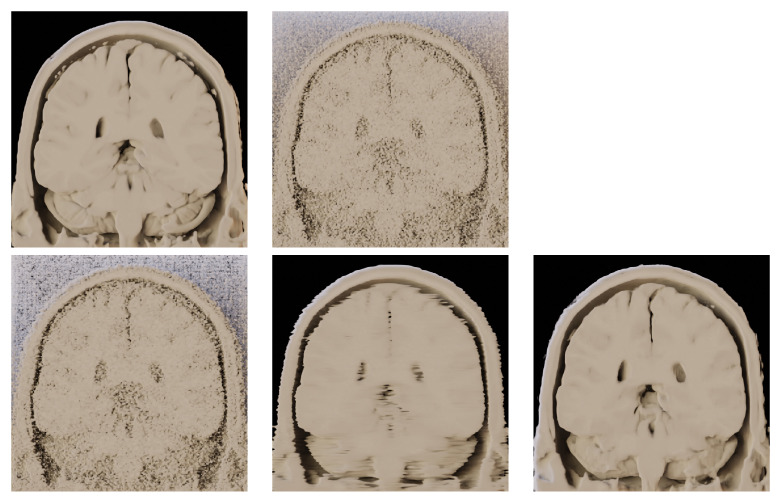
Application of volume rendering on the outputs of different denoising methods. BrainWeb dataset (181 × 181 × 181 voxels). Coronal view on the datastack from slice 92 to 181. (**top left**)—Original noise-less data. (**top right**)—Original data corrupted by 25% Gaussian noise. (**bottom left**)—RED-CNN. (**bottom middle**)—OIDN. (**bottom right**)— BM4D.

Since the methods for noise reduction in medical images mainly serve the purpose of further visual analysis, we have used the outputs of the previously mentioned methods and performed a volume rendering via path tracing on the fully denoised data stacks. The results can be seen in Figure 13.

## 4. Conclusions

We have shown how to parallelise one of the best-performing filtering techniques for denoising volumetric image data called BM4D. Its usage can be targeted mainly towards medicine, where large volumetric image data are frequently used. Our implementation can bring significant speed up in the total runtime of the filter. Based on our observation, we can achieve speed up from 200× to 300× compared to the original BM4D code version while running on multiple computing nodes (15 nodes). This is a reduction in processing time of between 99.5% and 99.7%. This brings the algorithm to very convenient usage since, on the largest dataset (512 × 512 × 779 voxels), the total runtime is 50.78 s instead of 3 h 59 m 16.30 s. Our parallelisation concept also allows full utilisation of the computing nodes. Either the node is equipped by CPUs only, MICs only or it is a computing node that combines them both. If a combination of CPUs and KNCs is used (full node utilisation on Salomon), it provides an enhanced performance of up to eight CPUs and eight KNCs against partial node utilisation (CPUs only). We have also compared BM4D denoising quality with specific DL methods and found that it can surpass the DL methods, which is proved by typical quality metrics and in the following volume rendering of denoised data.

In our future work, we plan to use the algorithm as a pre-processing step before volume rendering medical datasets by path tracing methods. This will allow us to obtain plausible visualisations of human body tissues in 3D.

## Figures and Tables

**Table 1:** Recommended parameters of the BM4D method that provide balanced performance in a wide range of noise variances.

Parameter		Stage	
		**Hard Thresholding**	**Wiener Filtering**
Cube size	L	4	5
Group size	M	32	
Step	$N_{step}$	3	
Search-cube size	$N_{S}$	11	
Similarity thr.	$\tau_{match}$	24.6	6.7
Shrinkage thr.	$\lambda_{4D}$	2.8	Not applicable

**Table 2:** Quantitative evaluation of different noise reduction methods in terms of denoising quality. Test performed on BrainWeb dataset (181 × 181 × 181 voxels), which has been corrupted by 25% Gaussian noise. Values computed as average over all slices in axial direction. Method marked as None provides measure between noise-less reference image and the image corrupted by the noise. The best values are in bold.

Method	Quality Measure		
	**PSNR**	**SSIM**	**RMSE**
None	13.663	0.617	0.208
RED-CNN	15.770	0.716	0.163
OIDN	22.235	0.902	0.078
BM4D	**23.547**	**0.921**	**0.067**

## Data Availability

The data are not publicly available due to the institutional GDPR policy.

## Abbreviations

The following abbreviations are used in this manuscript:

CT	Computed Tomography
MRI	Magnetic Resonance Imaging
DL	Deep Learning
HPC	High-Performance Computing
CPU	Central Processing Unit
MIC	Many Integrated Core
OpenMP	Open Multi-Processing
MPI	Message Passing Interface
NUMA	Non-Uniform Memory Access
KNC	Knights Corner
KNL	Knights Landing
